# The influence of pericardial fat upon left ventricular function in obese females: evidence of a site-specific effect

**DOI:** 10.1186/1532-429X-16-37

**Published:** 2014-05-23

**Authors:** Ning Hua, Zhongjing Chen, Alkystis Phinikaridou, Tuan Pham, Ye Qiao, Michael P LaValley, Sherman J Bigornia, Megan R Ruth, Caroline M Apovian, Frederick L Ruberg, James A Hamilton

**Affiliations:** 1 The Department of Physiology and Biophysics, Boston University School of Medicine, Boston, MA, USA; 2 The Division of Imaging Sciences and Biomedical Engineering, King’s College London, London, UK; 3 The Department of Biomedical Engineering, Boston University, Boston, MA, USA; 4 The Russell H. Morgan Department of Radiology and Radiological Sciences, Johns Hopkins Hospital, Baltimore, MD, USA; 5 The Department of Biostatistics, Boston University School of Medicine, Boston, MA, USA; 6 The Department of Endocrinology, Diabetes, and Nutrition, Boston University Medical Center, Boston, MA, USA; 7 The Department of Medicine, Section of Cardiovascular Medicine, Boston University School of Medicine, Boston, MA, USA; 8 The Department of Radiology, Boston University School of Medicine, Boston, MA, USA

**Keywords:** Cardiovascular magnetic resonance, Obesity, Pericardial fat

## Abstract

**Background:**

Although increased volume of pericardial fat has been associated with decreased cardiac function, it is unclear whether this association is mediated by systemic overall obesity or direct regional fat interactions. We hypothesized that if local effects dominate, left ventricular (LV) function would be most strongly associated with pericardial fat that surrounds the left rather than the right ventricle (RV).

**Methods:**

Female obese subjects (n = 60) had cardiovascular magnetic resonance (CMR) scans to obtain measures of LV function and pericardial fat volumes. LV function was obtained using the cine steady state free precession imaging in short axis orientation. The amount of pericardial fat was determined volumetrically by the cardiac gated T1 black blood imaging and normalized to body surface area.

**Results:**

In this study cohort, LV fat correlated with several LV hemodynamic measurements including cardiac output (r = -0.41, p = 0.001) and stroke volume (r = -0.26, p = 0.05), as well as diastolic functional parameters including peak-early-filling rate (r = -0.38, p = 0.01), early late filling ratio (r = -0.34, p = 0.03), and time to peak-early-filling (r = 0.34, p = 0.03). These correlations remained significant even after adjusting for the body mass index and the blood pressure. However, similar correlations became weakened or even disappeared between RV fat and LV function. LV function was not correlated with systemic plasma factors, such as C-reactive protein (CRP), B-type natriuretic peptide (BNP), Interleukin-6 (IL-6), resistin and adiponectin (all p > 0.05).

**Conclusions:**

LV hemodynamic and diastolic function was associated more with LV fat as compared to RV or total pericardial fat, but not with systemic inflammatory markers or adipokines. The correlations between LV function and pericardial fat remained significant even after adjusting for systemic factors. These findings suggest a site-specific influence of pericardial fat on LV function, which could imply local secretion of molecules into the underlying tissue or an anatomic effect, both mechanisms meriting future evaluation.

## Background

Recent studies of humans using a variety of approaches have suggested that regional fat distribution, rather than overall obesity plays an important role in cardiac modification [1-4]. Pericardial fat, a regional fat depot, is a metabolically active organ that secretes several bioactive proteins, including adiponectin, resistin, various inflammatory molecules, and is a rich source of free fatty acids (FFA) [5-8]. Pericardial fat could therefore exert local effects by multiple mechanisms on the underlying anatomic structures, including the heart and coronary arteries. Previous studies have linked excess pericardial fat to decreased cardiac indices, impaired diastolic filling or even congestive heart failure, and measures of cardiac structure such as left ventricular (LV) mass and left atrial size [9-11]. However, it is not clear whether the described associations between increased pericardial fat and adverse heart modifications are mediated by systemic effects caused by overall obesity or are a result of direct local interactions caused by the regional fat.

To explore which effect dominates LV modifications, we segmented and quantified fat covering the LV and the right ventricle (RV) separately to examine relationships of these specific fat depots to LV function. We hypothesized that if local effects dominate, pericardial fat surrounding the left ventricle would be associated more with measures of structure and function from the same chamber as opposed to the adjacent one. Since cardiovascular magnetic resonance (CMR) provides high spatial resolution and has the ability to perform 3D measurements, while maintaining adequate temporal resolution, it was chosen as the preferred imaging modality.

## Methods

### Subject recruitment

Female obese subjects (BMI ≥ 30 kg/m^2^, n = 60, age = 21-60 yrs) with no known cardiovascular disease were recruited from the Nutrition and Weight Management Center at Boston Medical Center with informed consent approved by the Intuitional Review Board of Boston University Medical Center. Subjects with atherosclerotic vascular disease, including those who have had stroke, myocardial infarctions, heart failure, prior percutaneous or surgical revascularization procedures, anginas, or peripheral arterial disease were excluded. In addition, subjects who weighed over 136 kg or had a waist circumference greater than 140 cm were excluded due to the size limitation of the scanner.

### Data acquisition

Subjects arrived for CMR in a fasting state (no food or liquid other than water consumed over the previous 8 hours). Height (to the nearest 0.1 cm), weight (to the nearest 0.1 kg), and waist circumference (in cm) were measured. BMI was calculated as body weight divided by height squared, and used as a marker of overall obesity. A fasting blood sample was collected by routine venipuncture, and the plasma levels of high-density lipoprotein (HDL), low-density lipoprotein (LDL), total cholesterol, triglyceride (TG), free fatty acids (FFA), fasting glucose, insulin, Interleukin-6 (IL-6), B-type natriuretic peptide (BNP), C-reactive protein (CRP), resistin and adiponectin were measured. Insulin resistance was calculated using the homeostatic model assessment (HOMA): HOMA_IR_ = (fasting insulin (mU/l) × fasting glucose (mg/dl))/405 [12]. The subjects underwent a CMR scan using a 3.0 T Philips Intera system (Philips Medical systems, Andover, MA). To quantify the pericardial fat volume, contiguous axial slices were obtained from the pulmonary bifurcation to the apex of the heart using an ECG gated, double inversion recovery 2D T1 weighted black blood (T1BB) sequence. The acquisition parameters were [9]: repetition time (TR) = 2 cardiac cycles, echo time (TE) = 13 ms, number of average (NEX) = 2, sense factor = 2.5, turbo factor (TSE) = 13, field of view (FOV) = 384 × 384 mm, matrix = 256 × 256, zero filled to 512 × 512, acquired resolution = 15 × 15 mm^2^, reconstructed resolution = 0.75 × 0.75 mm^2^, slice thickness = 4 mm. In order to assess cardiac function, short axis cine CMR was performed using a steady state free precession (SSFP) sequence with 8-11 s breath holds and ECG gating. Contiguous slices were obtained from the artrio-ventricular groove to the apex of the heart. The acquisition parameters were: TR = 3.0 ms, TE =1.6 ms, NEX = 1, sense factor = 1.5, FOV = 400 × 400 mm, matrix = 256 × 256, with 26–28 heart phases acquired per slice, slice thickness = 10 mm.

### Data analysis

**Figure 1:**
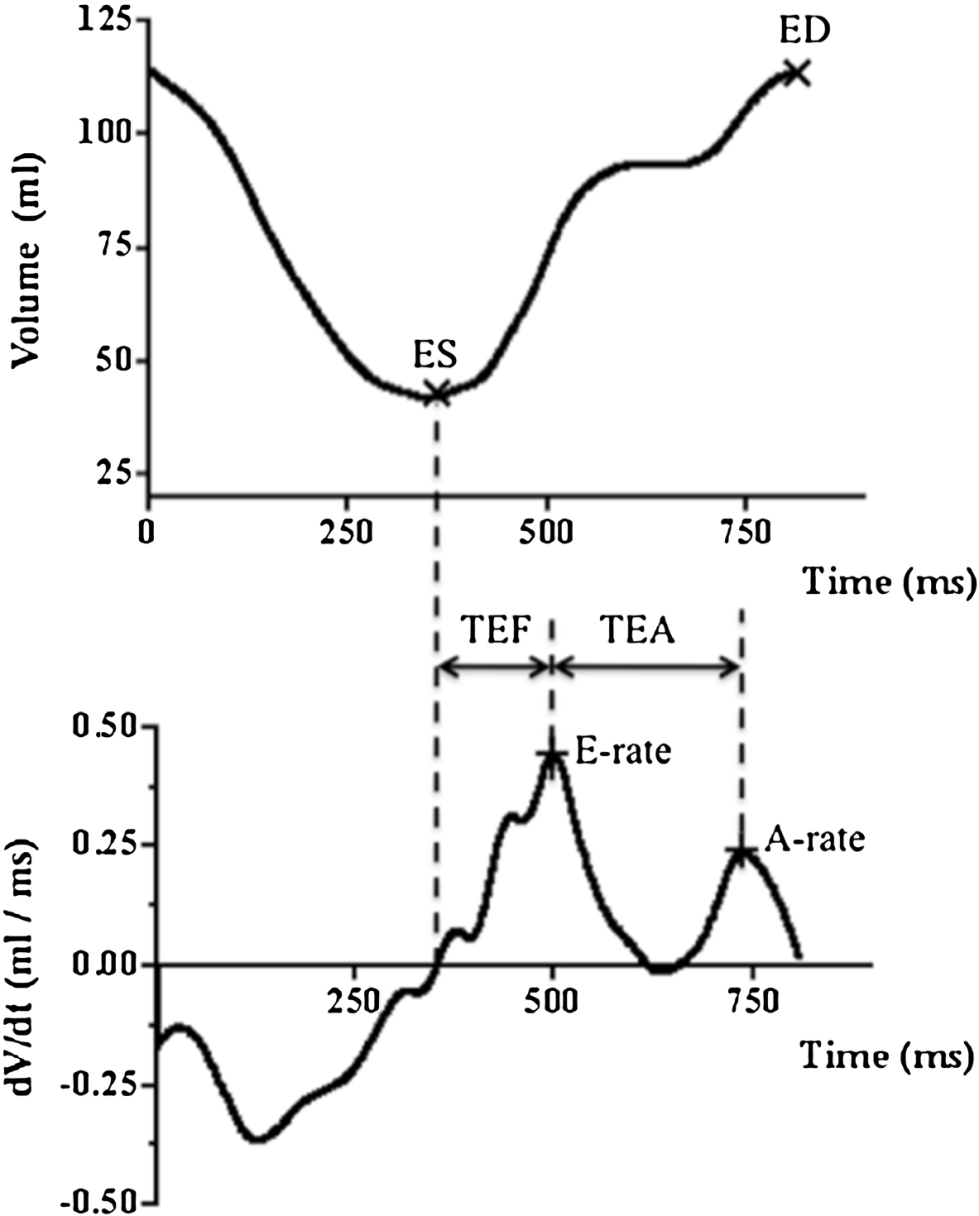
**Method for the calculation of left ventricular diastolic function.** Upper row, the volume-time curve of the left ventricle; bottom row, the first derivative curve of the volume-time curve. ES indicates the end of systole, and ED indicates the end of diastole. In the first derivative curve, the first appeared peak was defined as the early filling rate (E-rate) and the second peak as the late filling rate (A-rate). The time from the end of systole to E-rate was defined as the time to early filling (TEF). The time from the E-rate to the A-rate peak (TEA) was also obtained.

Pericardial fat was quantified using an in-house software programmed in Matlab (The MathWorks, Inc., Natick, MA). After images were corrected for inhomogeneity using a bias-field algorithm [13], pericardial fat segmentation was achieved by manually tracing the contours of pericardial fat on a slice-by-slice basis. For total pericardial fat, the superior and inferior boarders were defined as the bifurcation of the pulmonary artery and the apex of heart. For LV and RV fat, the atrio-ventricular groove was chosen as the anatomic marker to separate the atriums and ventricles, while the septum was used to separate the LV and RV. A threshold was chosen based on the histogram (between the 2nd and 3rd peaks of the histogram) in each slice, and the voxels with intensity above the threshold within each region-of-interest (ROI) were characterized as pericardial fat. Pericardial fat volume was calculated based on the Simpson Rule and then normalized to the body surface area (BSA) to compensate for the differences in heart size, and total, LV, and RV pericardial fat indices (TPI, LVFI, RVFI) were obtained. To assess inter-reader reproducibility, two independent observers measured pericardial fat. The interclass correlation coefficients (ICC) were excellent for pericardial fat measurements (ICC _LV fat_ = 0.94, ICC _RV fat_ = 0.89, ICC _total pericardial fat_ = 0.90).Cardiac function including left ventricular ejection fraction (EF), stroke volume (SV), and cardiac output (CO) were calculated using ViewForum (Philips, Andover, MA). Analysis was performed on contiguous short axis slices and summed up by Simpson’s technique. Diastolic function was determined based on the volume-time curve obtained from the previous step and processed using an in-house software (Matlab). The first derivative of the volume-time curve was obtained (Figure 1). The first peak that appeared was defined as the early filling rate (E-rate) and the second peak as the late filling rate (A-rate). The ratio of E/A was also calculated. The time from the end of systole to E-rate was defined as the time to early filling (TEF). The time from the E-rate to the A-rate peak (TEA) was obtained.

### Statistical analysis

Data are presented as mean ± SD. Comparisons between groups were performed using the student *t*-test. Associations between continuous variables were assessed using Pearson’s correlation. Multivariable adjusted linear regression was used to assess the significance of relation between the LV pericardial fat and LV function. Due to the limitation of our sample size, covariates included only BMI and the systolic blood pressure. A Two-tailed probability value *p* < 0.05 was defined as statistical significance.

### Terminology

Pericardial fat presented in the current study includes both epicardial fat (the lipid layer between the myocardium and the pericardium) and the fat external to the pericardium, as the two can generally not be separated in our measurements.

## Results

### Subject characteristics

**Figure 2:**
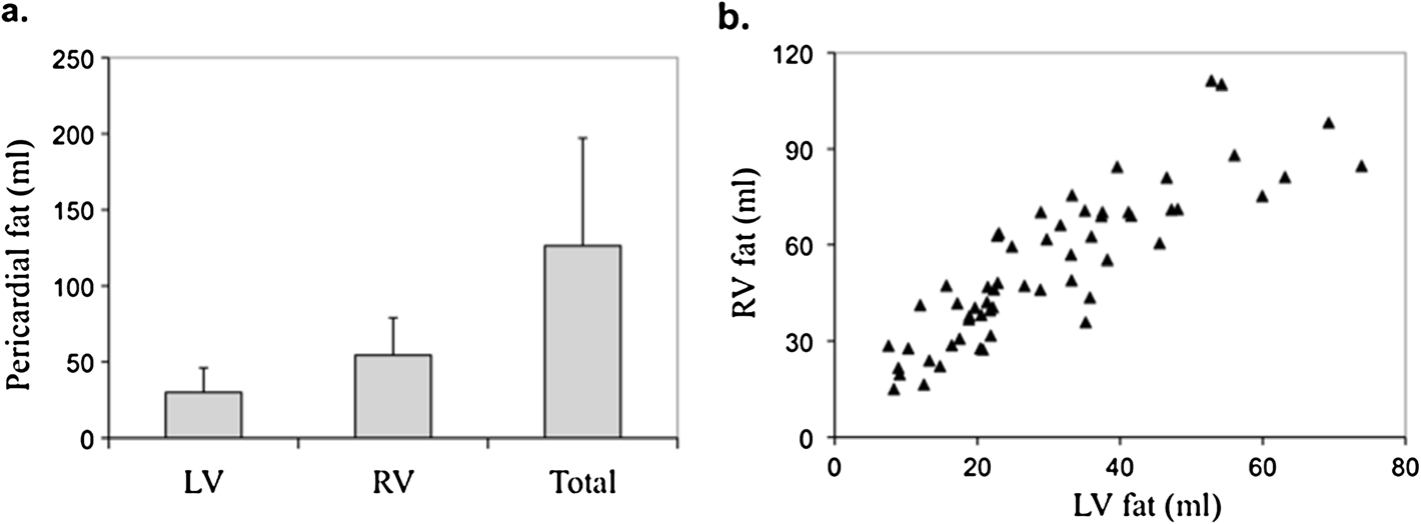
**Pericardial fat distribution. (a)** The average volume of pericardial fat was 126.1 ± 71.0 ml. More (p < 0.001) pericardial fat was observed around the right ventricle (54.4 ± 24.5 ml) than the left ventricle (29.9 ± 16.0 ml). **(b)** The fat depots surrounding the two ventricles are highly correlated in volume (r = 0.86, p < 0.001).

**Figure 3:**
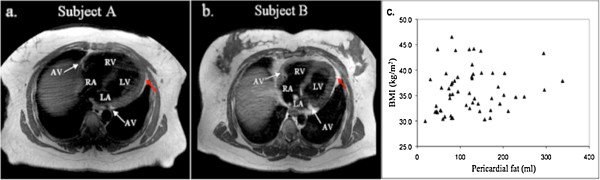
**The relationship between pericardial fat and body mass index.** Representative T1-black blood cardiac images shows that, as compared to the other subject, the one with the larger body mass index (BMI) has much less pericardial fat: **(a)**, A 36 year old subject, with BMI = 38.2 kg/m^2^, pericardial fat = 31 ml; **(b)**, A 49 year subject, with BMI = 31.9 kg/m^2^, pericardial fat = 213 ml; Red arrows point to pericardial fat. **(c)** Statistical analysis further showed that pericardial fat was not correlated with BMI in this study cohort (p = 0.14).

The study sample consisted of 60 obese females (BMI = 35.9 ± 4.6 kg/m^2^). Table 1 shows the clinical characteristics of our subjects. The average volume of pericardial fat was 126.1 ± 71.0 ml with more fat presented on the RV than the LV (Figure 2a, RV: 54.4 ± 24.5 ml; LV: 29.9 ± 16.0 ml; p < 0.001). Correlations of pericardial fat with plasma biomarkers were found significant for CRP level (LV fat: r = 0.38, p = 0.009; RV fat: r = 0.41, p = 0.006; total pericardial fat, r = 0.49, p < 0.001), but not for BNP, IL-6, resistin, adiponectin levels (all p > 0.05). The fat depots surrounding the two ventricles were highly correlated in volume (Figure 2b, r = 0.84, p < 0.001). Figure 3a-b shows the representative T1BB images of two subjects. Among the two, the more obese subject (BMI = 38.2 Kg/m^2^, Figure 3a) showed the less pericardial fat (31 ml), as compared with the other one (BMI = 31.9 Kg/m^2^, pericardial fat = 213 ml, Figure 3b). As in our previous study [9], the lack of association between local fat and overall obesity (BMI) was also observed at statistical level using Pearson correlation (Figure 3c, p = 0.14).

**Table 1:** Clinic characteristics of the total study sample

**Number of subjects**	**60**
Age, y	42.4 ± 12.0^a^
Body mass index, kg/m^2^	35.9 ± 4.6
Body surface area, m^2^	2.0 ± 0.17
Waist circumference, cm	105 ± 12
Systolic BP^b^, mmHg	123 ± 15
Diastolic BP, mmHg	79 ± 10
Total cholesterol, mg/dl	180 ± 37
Free-fatty acids, mmol/l	0.52 ± 0.23
Fasting glucose, mg/dl	102 ± 33
High density lipoprotein, mg/dl	48 ± 11
Low density lipoprotein, mg/dl	107 ± 31
Triglycerides, mg/dl	103 ± 53
HOMA_IR_^c^	3.31 ± 3.36

^a^Values are mean ± SD. ^b^BP indicates blood pressure. ^c^HOMA_IR_ indicates the insulin resistance calculated using the homeostatic model assessment (HOMA).

### Pericardial fat and LV function

Although LV and RV fat were highly correlated; they showed different associations with LV function (Table 2). LV CO and SV negatively correlated with LVFI (SV: r = -0.26, p = 0.05; CO: r = -0.41, p = 0.001), but not with RVFI (CO: p = 0.16; SV: p = 0.70) or TPFI (SV: p = 0.50; CO: p = 0.12). After excluding one outlier, LV fat also showed a stronger correlation with LV diastolic function as compared with RV fat. LV E-rate experienced a negative correlation with LVFI (r = -0.38, p = 0.01), but not with both RVFI (r = -0.24, p = 0.13) and TPFI (r = -0.28, p = 0.07). E/A was related to LVFI (r = -0.34, p = 0.03) and TPFI (r = -0.31, p = 0.04), but not RVFI (r = -0.24, p = 0.13). TEF positively correlated with LVFI (r = 0.34, p = 0.03), and weaker with RVFI (r = 0.30, p = 0.04) but not with TPFI (p = 0.07). No correlations were observed between LV A-rate, ejection fraction and the three of pericardial fat indices (all p > 0.05).

**Table 2:** Pearson Correlation Coefficients between Pericardial Fat and LV Function

	**LVFI**	**RVFI**	**TPFI**
SV	-0.26*	-0.06	-0.11
CO	-0.41*	-0.19	-0.21
EF	0.07	0.06	0.09
E-rate	-0.38*	-0.24	-0.28
A-rate	-0.06	-0.06	-0.01
E/A	-0.34*	-0.23	-0.31*
TEF	0.34*	0.30*	0.28

*, p < 0.05.

### LV function and systemic factors

Pearson correlation showed LV function did not correlate with systemic inflammatory markers or plasma adipokines, such as CRP, BNP, IL-6, resistin and adiponectin (all p = NS). To control for potential systemic confounders, the relations between LV fat and LV function was further accessed by the multiple linear regression. After adjusting for BMI and the systolic blood pressure, LV fat remained significantly correlated (Table 3) to the decreases of left ventricular SV (β = -0.23, p = 0.05), CO (β = -0.33, p = 0.002), E-rate (β = -0.28, p = 0.05) and the prolonged TEF (β = 0.36, p = 0.03). Borderline significance was observed between LV fat and E/A (β = -0.24, p = 0.10) after the multivariable adjustment.

**Table 3:** **Multivariable**-**Adjusted Regressions for LV Pericardial Fat and LV Function**

	**Standardized β**	**P**
SV	-0.23	0.05
CO	-0.33	0.002
E-rate	-0.28	0.05
E/A	-0.24	0.10
TEF	0.36	0.03

## Discussion

### Principal findings

To our knowledge, this is the first report indicating that the relations of LV function to LV fat are stronger than RV fat, and remain significant after the multivariable adjustment. Meanwhile, no association was observed between LV function and systemic factors including plasma inflammatory markers and adipokines. These findings suggest that pericardial fat may exert local endocrine or anatomic effects on LV function.

Obesity, defined as BMI > 30 kg/m^2^, has been well established as an independent risk factor for cardiovascular diseases [14,15]. However, some recent studies suggested that regional fat distribution rather than the total amount contributes to obesity-related adverse modifications [16-21]. Among various regional fat depots, pericardial fat is of particular interest and has been proposed as a superior indicator of cardiac function because of the anatomic vicinity. Iacobellis et al. [10] studied 30 morbidly obese subjects and compared with 20 lean control volunteers using 2D echocardiography. They found an increase in the epicardial fat thickness strongly correlated with the decrease of LV diastolic function. Ruberg et al. [9] reported the negative correlations between pericardial fat and LV CO/SV in an obese group with metabolic syndrome. Both studies suggested local interactions between the regional adipose and LV function, but without ruling out the influence from systemic effects caused by overall obesity. It became particular interesting when Fox et al. performed a large population study [22] in 2009. They reported that pericardial fat correlated with LV EDV and atrial dimension, but these correlations did not hold after the multivariable adjustment for overall adiposity, and suggested that systemic influences might override local effects. This study [22] did not examine the specific LV function as the previous two [9,10]. However, the same rationale might apply, which is the previously observed relations between the local fat and cardiac function [9,10] may actually be the carryover effect from overall obesity.

To address this issue, we examined the relations between pericardial fat and LV function from different aspects and found novel results for our obese subjects (BMI: 30.0-46.5 kg/m^2^). Firstly, dissociation was observed between pericardial fat and BMI, indicating that regional fat accumulation can deviate from total body fat accumulation. In other words, it is possible to find obese subjects with “normal” amount of pericardial fat, which may not carry the pathologic influences from distal fat if local effects dominate. Secondly, LV function did not correlate with serum biomarkers, including IL-6, CRP, BNP, resistin and adiponectin. These biomarkers have been found elevated/decreased in obese people, and linked to the increased inflammatory stress, which in turn could be detrimental to cardiac function [23-27]. The lack of correlation between LV function and these serum markers suggests if endocrine effects of adiposity exist, it is likely come majorly from the local fat instead of distal fat accumulation. Finally, we have refined the measurement of the local fat, by segmenting pericardial fat overlaying different ventricles, then correlating to LV function separately. LV fat showed a consistent stronger correlation pattern to LV function, as compared with RV fat in our study cohort. In particular, the hemodynamic function of LV negatively correlated with LV but not RV fat. The increase of LV fat was also related to significant and stronger reductions of LV diastolic function, including the decreases of E-rate, E/A, and the prolonged TEF, as compared with RV fat. Moreover, most of the significant relations between LV fat and LV function still remained significant even after adjusting for two major reported confounders: BMI and the blood pressure. Since the left and right ventricles are under the same systemic influences, the additional associations between LV function and LV fat compared to RV fat suggests the local effects of pericardial fat on LV function. Although the detailed mechanisms remain to be clarified, it is possible that LV fat exerts direct anatomic pressure or elaborates FFA, resistin and other adipokines through coronary arteries, which branch off and feed the left and the right ventricles separately. In such a case, LV fat would show a stronger correlation to LV function as compared to RV fat, which does not contact or supply blood to the left ventricle directly.

In this study cohort, subjects with severe cardiac dysfunction were excluded, and late stage diastolic dysfunction with pseudonormal diastolic pattern is not likely to occur. Hence, it is reasonable to believe only linear relationship may exist between LV diastolic function and pericardial fat. In this study cohort, LV diastolic indices showed correlations with RV fat, but to a lesser extent as compared with LV fat. This observation may not necessarily suggest that RV fat has a direct influence on LV relaxation, as it could result from the strong correlation between the two fat depots in volume. This evidence implies that LV diastolic function may be impaired to a greater extent by the presence of the local adipose tissue. Various studies [28,29] have reported that subjects with impaired diastolic filling may still have normal contractility, suggesting that the development of diastolic abnormalities generally precedes the systolic dysfunction. Similarly, our data showed that LV fat strongly correlated with several diastolic indices; however, no correlation was observed between pericardial fat and the contractile measurement, ejection fraction. Several mechanisms have been proposed to explain the early influences on LV diastolic function. Fox et al. [22] suggested a mechanical role of pericardial fat in diastolic dysfunction. The compression force generated by local adiposity might affect LV relaxation and induce atrial dilation. In addition, the adipose tissue around the heart is a metabolically active organ that secretes FFA, resistin and other adipokines, which are strongly linked with insulin resistance [5,30]. Insulin resistance may affect ventricular relaxation by impairing the myocardial actin-myosin dissociation through increased intracellular Ca^2+^levels, because of reduced activity of Na^+^/K^+^ ATP-ase in the plasma membrane or decreased reuptake of Ca^2+^ into the sarcoplasmic reticulum [31,32].

### Strengths and limitations

The 3D volumetric assessment of pericardial fat and LV function provides an advantage over previous studies that used the fat thickness for similar assessments. Additionally, by considering left ventricular fat separately from the adjacent pericardial fat, we were able to more accurately describe the relationship between cardiac function and regional fat depots. Some limitations warrant discussion. In particular, epicardial fat is more proximal to the myocardium and has a different metabolic activity than the fat outside the pericardium [33], which theoretically would make epicardial fat a more interesting target to investigate. We were unable to distinguish the epicardial fat in all subjects, so the observed associations are partly contributed by the fat outside the pericardium. However, a previous study showed that epicardial and pericardial fat depots are highly correlated in volume (r = 0.92, p < 0.0001) in a group of randomly selected 159 subjects [34]. Similarly, we were able to separate the two fat depots in images of 13 subjects, for whom epicardial fat was strongly correlated with pericardial fat (r = 0.92, p < 0.001, the 95% confidence interval of r: 0.76 ~ 0.98). This suggests that in our subjects, epicardial fat may have a similar relationship as seen between pericardial fat and cardiac function. Also, compared to echocardialography studies, cine- CMR has a relatively low temporal resolution of 28-30 ms, which does not allow capture of some important parameters that measure the delay in diastolic dysfunction, such as isovolumic relaxation time (IVRT). In addition, the current study was cross-sectional in design and cannot infer causality. Furthermore, the size limitation of our closed bore CMR scanner necessitated exclusion of male subjects, who tend to have large waist circumferences. Male subjects with larger BMI will be an important group for future studies with appropriate CMR scanners. Finally, obstructive sleep apnea or female specific medical conditions, such as polycystic ovarian syndrome, menopause and may have an influences on the cardiac outcomes. These more complicated conditions were not considered in the current work and would require studies with larger sample sizes.

## Conclusions

Variation in LV hemodynamics and diastolic function, independent of body size or blood pressure, was observed that related specifically to the volume of pericardial fat overlaying the LV. Variation did appeared to associated to a lesser degree with fat overlying the RV, but was independent of serum inflammatory markers or adipokines in this healthy but obese population. These findings provide insight into a possible mechanism by which metabolic disease, and pericardial fat accumulation, might negatively impact cardiac function.

## Competing interests

The authors declare that they have no competing interests.

## Authors’ contributions

NH conceived the study, participated data acquisition, data analysis and drafted the manuscript; ZJC, QY and AP participated CMR data acquisition; TP participated in data analysis; MPL performed the statistical analysis; SJB, MRR cordinated the study and participated in biological data acquisition; CMA, FLR and JAH participated in the study design. All authors read and approved the final manuscript.
